# Red-Shifted Firefly Luciferase Optimized for *Candida albicans In vivo* Bioluminescence Imaging

**DOI:** 10.3389/fmicb.2017.01478

**Published:** 2017-08-03

**Authors:** Stephane Dorsaz, Alix T. Coste, Dominique Sanglard

**Affiliations:** Institute of Microbiology, University of Lausanne Lausanne, Switzerland

**Keywords:** *Candida albicans*, luciferase, mice, antifungal agents, imaging techniques

## Abstract

*Candida albicans* is a major fungal pathogen causing life-threatening diseases in immuno-compromised patients. The efficacy of current drugs to combat *C. albicans* infections is limited, as these infections have a 40–60% mortality rate. There is a real need for novel therapeutic approaches, but such advances require a detailed knowledge of *C. albicans* and its *in vivo* pathogenesis. Additionally, any novel antifungal drugs against *C. albicans* infections will need to be tested for their *in vivo* efficacy over time. Fungal pathogenesis and drug-mediated resolution studies can both be evaluated using non-invasive *in vivo* imaging technologies. In the work presented here, we used a codon-optimized firefly luciferase reporter system for detecting *C. albicans* in mice. We adapted the firefly luciferase in order to improve its maximum emission intensity in the red light range (600–700 nm) as well as to improve its thermostability in mice. All non-invasive *in vivo* imaging of experimental animals was performed with a multimodal imaging system able to detect luminescent reporters and capture both reflectance and X-ray images. The modified firefly luciferase expressed in *C. albicans* (Mut2) was found to significantly increase the sensitivity of bioluminescence imaging (BLI) in systemic infections as compared to unmodified luciferase (Mut0). The same modified bioluminescence reporter system was used in an oropharyngeal candidiasis model. In both animal models, fungal loads could be correlated to the intensity of emitted light. Antifungal treatment efficacies were also evaluated on the basis of BLI signal intensity. In conclusion, BLI with a red-shifted firefly luciferase was found to be a powerful tool for testing the fate of *C. albicans* in various mice infection models.

## Introduction

Fungal pathogens can cause a wide variety of diseases ranging from superficial infections to life- threatening invasive infections. It is believed that invasive infections cause at least 1.5 million deaths per year. *Candida* spp., and especially *C. albicans*, contribute to ~25% of all life-threatening invasive diseases, despite antifungal treatments (Brown et al., 2012). These numbers highlight the need to better understand how *C. albicans* infects its host, and to propose better therapeutic alternatives.

Investigating *C. albicans* pathogenesis requires the use of animal infection models which mimic the human host. The testing of alternative therapies to combat *C. albicans* infections also necessitates these models. Classically, animal models of *Candida* infections use mammalian hosts (mice, rabbit, rat; Coste and Amorim-Vaz, 2015). The use of more simple insect models such as *Galleria mellonella* has been also undertaken (Amorim-Vaz et al., 2015).

Animal model infections face several challenges when following the progression of a fungal infection. Fungal species such as *C. albicans* propagate in different tissues over time. In the case of an invasive infection performed by intravenous injection, *C. albicans* reaches several organs (liver, spleen, kidneys) over time and eventually accumulate in the kidneys (MacCallum and Odds, 2005). In the case of oropharyngeal candidiasis (OPC), superficial and underlying epithelial tissues are affected without systemic spread of the fungus (Mosci et al., 2013; Trautwein-Weidner et al., 2015). One other issue is the necessity to evaluate the infection by determination of fungal burden, which has traditionally involved euthanasia. After euthanasia, specific tissues are chosen, homogenized, and plated onto corresponding growth media to enumerate viable fungal cells. Several alternatives to this labor-intensive classical procedure have been proposed recently (Sturtevant, 2009; Zhang et al., 2013), one of which allows the fate of fungal cells in living animals to be followed in real time, using so called *in vivo* imaging (Brock, 2012).

In order to perform *in vivo* imaging, fungal cells need to be recognized by imaging instruments. In the recent years, bioluminescent imaging (BLI) has been the method of choice for recording the presence of fungal elements in living tissues, as it is an imaging modality that typically has excellent Signal to Noise ratios (SNRs). It is widely known that bioluminescence can be emitted by any one of several light production systems in fungi. These include the *Gaussia princep*, the firefly and the click beetle luciferases (Enjalbert et al., 2009; Jacobsen et al., 2014). Each system uses codon-optimized gene and constitutive high expression systems. *G. princeps* luciferase uses coelenterazine as substrate and oxygen as co-factors to emit light, while the click beetle and firefly luciferases use luciferin and ATP as co-factors. Further, given that the *G. princeps* luciferase is naturally a secreted protein, a modified version has been expressed so that it will localize at the *C. albicans* cell wall through the addition of a GPI-anchor (Enjalbert et al., 2009). In contrast, firefly luciferase can only be an intracellular enzyme, since it needs ATP produced by internal metabolic activity for light emission. It is also widely recognized that *in vivo* BLI signals scatter and tissue absorption is a function of bioluminescent light spectral properties. While the emission peak of *G. princeps* luciferase is at 480 nm, firefly luciferase has an emission peak at around 560 nm. Additionally, two forms of click beetle luciferase exist, one emitting in the green light at 537 nm and one emitting in the red light at 613 nm. However, these two luciferases are still only used *in vitro* (Kapitan et al., 2016).

Given the above mentioned diversity of available fungal luciferase systems, the selection of which luciferase option to use for a given animal model is an important decision. Since firefly luciferase has a light emission peak that extends toward red light, it has a decisive advantage over *G. princeps* luciferase light emission which is limited to blue light. In the context of animal tissues, the range of light peak emission is important since blue light is more absorbed by host tissues than is red-shifted light. This phenomenon is clearly illustrated by two studies that each uses a different luciferase (Enjalbert et al., 2009; Jacobsen et al., 2014). First, in the study of Enjalbert et al. (2009), *C. albicans* strains expressing GPI-anchored *G. princeps* luciferase were used, and no satisfactory correlation was obtained between the fungal burden and the light emitted following mice systemic infection. In contrast, the *C. albicans* codon-optimized firefly luciferase in a second study demonstrated a good correlation between light emission and fungal burden in systemic infection (Jacobsen et al., 2014). Firefly luciferase is, therefore, considered to be the BLI reporter of choice when following bioluminescent cells in infected mammals. In the study of Jacobsen et al. (2014), the limit of luminescence detection corresponded to about 3,000 cells per kidney, which represents an already important amount of cells infecting the tissues. It is known that firefly luciferase can be modified in order to improve its spectral properties as well as enzymatic and thermal stability properties. Such modifications have been exploited to increase sensitivity of BLI in different type of animal cells expressed in animal models. Until now, no further improvement of firefly luciferase has been implemented in *C. albicans* cells.

In this work, we undertook the modification of firefly luciferase in order to improve the detection limits of *C. albicans* cells in infected mice. We used here two different *C. albicans* infection models (systemic and OPC infections) and demonstrate that the modified firefly luciferase performs better than the wild type version. In addition, we used here a novel high sensitive *in vivo* imaging system coupled with the specific needs of containment of infected animals.

## Materials and methods

### Strains, plasmids, and growth conditions

*Escherichia coli* DH5α was used as a host for plasmids propagation. To grow DH5α, LB broth medium was used and when necessary, supplemented with ampicillin (0.1 mg/ml). Cultures of *E. coli* were incubated at 37°C under constant agitation (220 rpm) for 16–20 h. For plate cultures, 0.7% BactoTM Agar (Brunschwig, Switzerland) was added.

*C. albicans* were grown in complete YEPD medium (1% Bacto peptone [Difco Laboratories, Basel, Switzerland], 0.5% yeast extract [Difco] and 2% glucose [Fluka, Buchs, Switzerland]) at 30°C under constant agitation (220 rpm). For growth on plates, 2% BactoTM Agar was added to the medium. Yeasts were transformed by a lithium-acetate procedure with slight modifications as previously described (Sanglard et al., 1996).

For the construction of the red-shifted luciferase, two rounds of mutagenesis were performed by PCR. First, two mutations (S284T and L295F) were introduced to allow peak emission red-shifting. For this purpose, two overlapping PCR were performed on pACT1-CaLucOpt (Jacobsen et al., 2014) using the Phusion® high-fidelity Taq polymerase (NEB, Switzerland). The first PCR was carried out with the primers Hind_LUCopt (GCAAGCTTAAAATGGAAGATGCTAAGAACA) and LUCopt_MUT1_R (AAAGTAGATTTAGCCAAGAAAGAGAACAAAGTTGGAACCAACAAAGCAGTTTGAATCTTG). The second PCR was performed with primers LUCopt_MUT1_F (CAAGATTCAAACTGCTTTGTTGGTTCCAACTTTGTTCTCTTTCTTGGCTAAATCTACTTT) and Nhe-LUCopt (GCAAGCTAGCCTATACAGCAATTTTACCAC). The primers LUCopt_MUT1_F and LUCopt_MUT1_R are overlapping and contained the mutations. Next, a third PCR was performed to fuse the two previous PCR products using 10 ng of each PCR product as template and the two external primers (Hind_LUCopt and Nhe-LUCopt). The final product was cloned after *Hind*III and *Nhe*I digestion in pACT1-GFP (Barelle et al., 2004), thus yielding pDS1901. A second round of mutagenesis was performed to introduce two mutations (T214A and A215L). The two PCR were performed on pDS1901. The first and second PCR used, respectively, the primer pair Hind_LUCopt and LUCopt_MUT2_R (GTGAGAGAATCTAACACACAAAGCTCTGTGTGGCAAAGCAAC) and the primer pair LUCopt_MUT2_F (GTTGCTTTGCCACACAGAGCTTTGTGTGTTAGATTCTCTCAC) and Nhe-LUCopt. The primers LUCopt_MUT1_F and LUCopt_MUT1_R are overlapping and contained the mutations. A last PCR was performed as above with primers Hind_LUCopt and Nhe-LUCopt and the final product was cloned in pACT1-GFP to yield pDS1902.

The initial plasmids pACT1-CaLucOpt and pDS1902 were digested by *Stu*I and transformed as described above in CAF4-2 (Fonzi and Irwin, 1993) to yield DSY4824 (expressing Mut0) and DSY4823 (expressing Mut2), respectively. To express Mut2 in another *C. albicans* strain (strain 101, a gift from S. Leibundgut, University of Zürich), a plasmid (pDS1928) containing the *SAT1* dominant marker was used (Reuss et al., 2004). pDS1928 was constructed from pDS1904, which contained the *SAT1* resistance marker in CIp10 (Murad et al., 2000). A 3.5 kb *Xho*I-*Bam*HI fragment from pDS1902 containing Mut2 was inserted in pDS1904 to yield pDS1928. This plasmid was linearized by *Stu*I for transformation into strain 101, yielding DSY4976. Five clones of each transformation were checked for luminescence signal and those with best signals were selected (data not shown).

### *In vitro* luciferase assay

For *in vitro* cells suspension assays, *C. albicans* strains were grown overnight in YEPD. Each strain was subsequently diluted 100-fold in 3 ml of YEPD medium and grown to a concentration of 1.5 × 10^7^ cells/ml. Cells were then washed twice in phosphate-buffered saline (PBS: 137 mM NaCl, 2.7 mM KCl, 10 mM Na_2_HPO_4_, 1.8 mM KH_2_PO_4_) and resuspended in 3 ml of PBS. Cells concentration was measured using a spectrophotometer. Cells were next centrifuged and resuspended in 2 × RPMI (Sigma, Switzerland) buffered at pH 7 or 4.6 with MOPS at 10^8^ cells/ml. Fifty microliters of the cells suspension were then loaded on a 96-well plate (half-area, black, COSTAR). Sixty microliter of D-luciferin (Biosynth, Switzerland) resuspended in H_2_O at a concentration of 1.6 mg/ml was injected automatically into the wells, followed by an orbital shacking of 3 s before recording luciferase activity (RLU: Relative Luminescent Unit) using a 96-well plates luminometer (FLUOstar Omega, BMG Labtech). Filters available for spectral analysis were at 460, 520, 530, 550, 580, 620, and 675 nm.

### Mice infection and antifungal therapy

All animal experiments were performed at the University Hospital Center of Lausanne with approval through the Institutional Animal Use Committee, Affaires Vétérinaires du Canton de Vaud, Switzerland (authorization n° 1734.4), according to decree 18 of the federal law on animal protection. For all mice experiments, female BALB/c mice (7 weeks old; Charles River France) were housed in ventilated cages with free access to food and water. They were controlled and scored daily for health status. Score of health was based on ability to heat and drink, to move, on fur aspect, on body weight and on body temperature (1 to 3 points for each criteria, where 1 corresponds to “healthy” and 3 to “strongly altered”). Healthy mice had a score of 5 points. Mice with a score above 10 or with a body weight loss equal or above 25% or body temperature below 35.5°C were immediately euthanized. The *C. albicans* strains were grown in individual tubes for 16 h under agitation at 30°C in YEPD medium. Each strain was subsequently diluted 100-fold in YEPD medium and grown overnight under agitation at 30°C. Overnight cultures were washed twice with PBS and resuspended in 5 ml PBS.

### Systemic infection

The concentration of each *C. albicans* culture was measured through optical density, and each strain was diluted to 2 × 10^6^ cells/ml. Mice were injected through the lateral tail vein with 250 μl of the cell suspension. For drug treatments, mice were injected intraperitoneally daily starting 24 h post-infection (p.i.) with 100 μl of fluconazole (FLC: 10 mg/kg; Toronto Research Chemicals) or 100 μl of caspofungin (CAS: 1 mg/kg; Cancidas® Merck, Switzerland). For determination of fungal burden, the kidneys of euthanized animals were removed, homogenized in sterile 0.05% NP40 in water and serial dilutions were plated on YEPD agar.

### Oropharyngeal infection

Immunocompetent mice were infected sublingually with 2.5 × 10^6^ CFU *C. albicans* yeast cells using a cotton ball as described (Schönherr et al., 2017). For determination of fungal burden, the tongue of euthanized animals was removed, homogenized in sterile 0.05% NP40 in water and serial dilutions were plated on YEPD agar. For drug treatment, FLC was diluted in 0.5% hydroxyethyl-cellulose (Sigma) and 30 μl of the preparation was administered under isoflurane anesthesia with 200 μl pipette tips under the tongue daily starting 24 h p.i. (10 mg/kg/day).

#### *In vivo* imaging and quantification of bioluminescence

*In vivo* imaging was performed on a Bruker in-vivo Xtreme II (Bruker BioSpin MRI GmbH, Ettlingen, Germany). In a laminar flow biosafety cabinet, 5 mice were generally anesthetized with 3% isoflurane in an induction chamber, and then placed in a Sealed Optical Imaging (OI) Tray, ventilated with 2.5% isoflurane. Once the animals were correctly placed, the Sealed OI Tray was disconnected from its anesthesia ventilation in the biosafety cabinet, and immediately placed into the in-vivo Xtreme II, where it was connected to 2.5% isoflurane ventilation. Animal's body temperature was managed by feedback regulated controlled air flow and the set to 37°C. BLI camera settings were: field of view (FOV) of 19 × 19 cm, 8 × 8 binning and 5 min of exposure time. X-ray imaging camera settings were: FOV of 19 × 19 cm and 5 s of exposure time. D-luciferin, a substrate for firefly luciferase, was injected intraperitoneally (100 μL D-luciferin, at 18 mg/ml, Biosynth, Switzerland) 10 min before luminescence imaging. The process of animal handling and image acquisition is presented in a movie (Supplementary Material). In addition to the intraperitoneal D-luciferin injection in the OPC model, 10 μl of 1.5 mg/ml D-luciferin was deposited under the tongue of the mouse prior to image acquisition.

A region of interest (ROI) template of fixed size for each kidney was used to ensure that areas of identical size were measured in different animals. Background was measured for each image and subtracted from the obtained signals. Value of luminescence (photons/s/mm^2^) of a given animal corresponds to the mean of the average luminescence of the two kidneys. ROI in the OPC model correspond to the head region of mice.

For the spectral shift assay, different emission filters were selected on the Bruker in-vivo Xtreme II machine at 535, 600, 700, 750, 790, and 830 nm, each with a bandpath of ~ ±20 nm.

## Results

### Design of a red-shifted *C. albicans* optimized firefly luciferase

**Figure 1:**
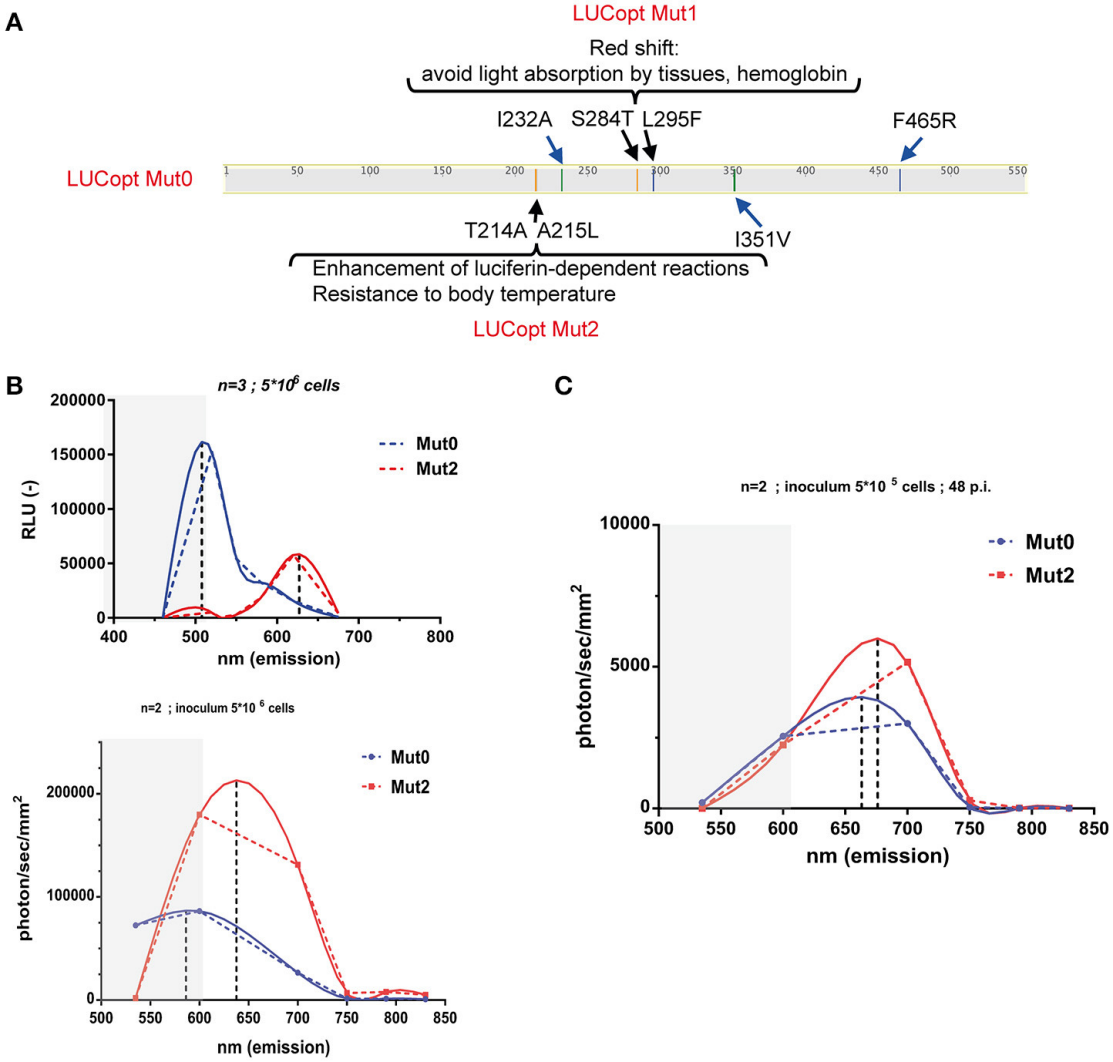
Red shifting of firefly luciferase. **(A)** Schematic representation of mutations in firefly luciferase and their effects. **(B)** *In vitro* spectral analysis of luciferases. *C. albicans* cells expressing Mut0 and Mut2 were grown to logarithmic phase and suspended in PBS as described in Material and Methods. Spectra were recorded with a luminometer (upper graph) or with in-vivo Xtreme II (lower graph) and analyzed with Graph Prism. Dotted and full lines represent direct connections between data points and data curve fitting, respectively. Gray zones represent data points not covered by the emission filters of the in-vivo Xtreme II instrument. Data were obtained with triplicate cultures of *C. albicans* expressing each Mut0 or Mut2 (5 × 10^6^ cells). **(C)** *In vivo* spectral analysis of luciferases. Mice infected with 5 × 10^5^ cells were subjected to spectral analysis at 48 p.i. with in-vivo Xtreme II, and obtained data were analyzed with Graph Prism.

Taking advantages of a *C. albicans* codon-adapted firefly luciferase (pACT1-CaLUCopt) reporter gene (Jacobsen et al., 2014), an optimization of this luciferase was undertaken to improve its properties. We were especially interested to improve *in vivo* light emission and tissue penetrance. We first introduce two mutations in CaLUCopt (S284T and L295F). S284T is known to result in a red-shifting in light emission, while L295F contributes to thermal stability (Branchini et al., 2005, 2007; Figure 1A). The rationale behind this idea was to reduce light absorption by tissues and hemoglobin, as reported (Papon et al., 2014). In a second round of mutations, two other substitutions were introduced (T214A and A215L) that were known to confer enhancement of luciferin-dependent reactions and a better resistance to body temperature as described (Branchini et al., 2007).

*C. albicans* transformants with the original and the optimized luciferase were obtained (designated as Mut0 and Mut2, respectively) and their light emission spectra analyzed first *in vitro* with whole cells on a standard laboratory luminometer with seven emission filters covering a 460–675 nm range. Firefly luciferase was detected in cell extracts from the *C. albicans* transformants in similar amounts (Figure S1). As shown in Figure 1B (upper panel), Mut0 showed a dual peak emission profile with a major peak at around 500 nm. In contrast, Mut2 exhibited a clear shift toward the red spectrum with a peak at about 625 nm. Similar *in vitro* measurements were conducted using the bruker in-vivo Xtreme II imaging platform, containing five filters spanning from 520 to 850 nm. The measured emission spectra profiles were similar to those obtained with a luminometer, except that the major peak of Mut0 emission could not be recorded. This instrument is dedicated to *in vivo* imaging and since wavelengths shorter than 550 nm are absorbed by host tissues, it does not record light emitted below 520 nm. Thus, it can be expected that Mut2 light emission could be better captured by the instrument camera, while an important portion of Mut0-emitted light may remain entrapped by mammalian tissues and is not recorded by the instrument dedicated to *in vivo* red-near infrared (NIR) imaging. In order to assess this assumption, emission spectra were next measured *in vivo* in mice infected with either *C. albicans* cells harboring Mut0 or Mut2 (inoculum 5 × 10^5^ CFU/mouse) at 48 h p.i.. This infection time allows the establishment of the *C. albicans* infection at the site of the kidneys. After spectral analysis, we could observe light emission peaks at higher wavelength for Mut2 as compared to Mut0 (Figure 1C). In addition, both luciferases displayed a peak emission shifted to higher wavelengths as compared to *in vitro* emission (Figure 1B, lower panel and Figure 1C), confirming that light in the green-blue region is entrapped by mammalian tissues and not recorded, as expected.

### Comparison of sensitivity of both luciferases *In vivo*

**Figure 2:**
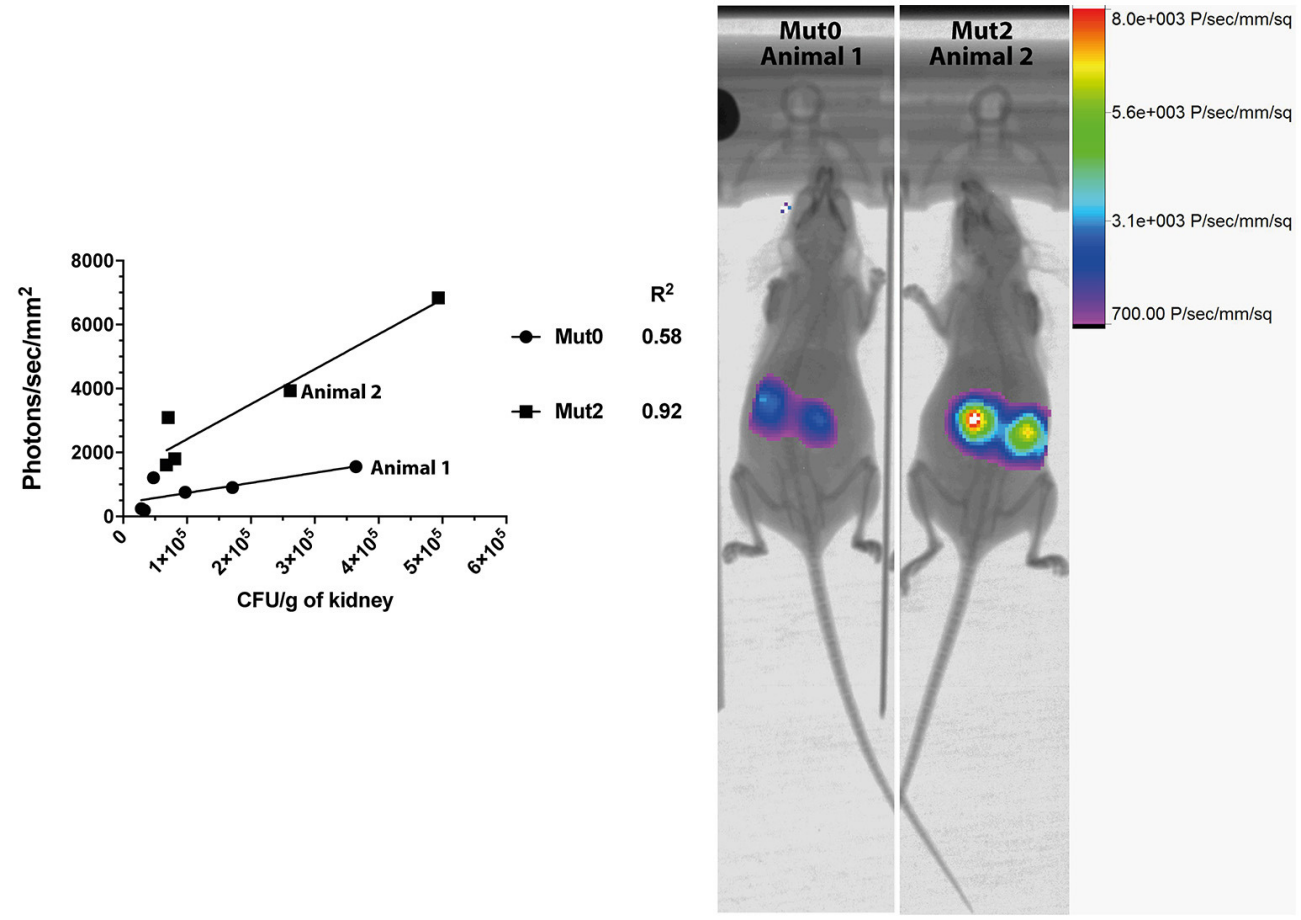
Comparison of *in vivo* light emission from luciferases expressed in *C. albicans*. Luminescence signals obtained from mice kidneys infected with *C. albicans* expressing either Mut0 or Mut2 were plotted against obtained CFU values. Corresponding X-ray and bioluminescence captures are shown at 48 h p.i. (right panel) and highlight differences of luminescence between Mut0 and Mut2. Luminescence data are means of two kidneys ROI values from individual animals. X-ray and luminescence overlay images of animals 1 and 2 correspond to data points plotted and indicated in the left panel.

We next addressed the performance of Mut2 in parallel to Mut0 in the invasive model of infection in mice. After acquisition of total emitted bioluminescence in mice in the region of the kidneys, fungal burdens in these organs were obtained and were compared to the signals emitted by both luciferase types (Figure 2). Signal intensities were about 3.7-fold higher for Mut2 as compared to Mut0 (median of 828.9 and 3,089 photons/s/mm^2^, respectively; *p* < 0.005, Mann-Whitney test). In addition, the Mut2 luminescent signal correlated better with CFU than Mut0 (R^2^ 0.92 vs. 0.58, Figure 2). These data clearly establish Mut2 firefly luciferase as a more sensitive *in vivo* luminescence reporter for *C. albicans*.

### *In vivo* dose response using the red-shifted firefly luciferase

**Figure 3:**
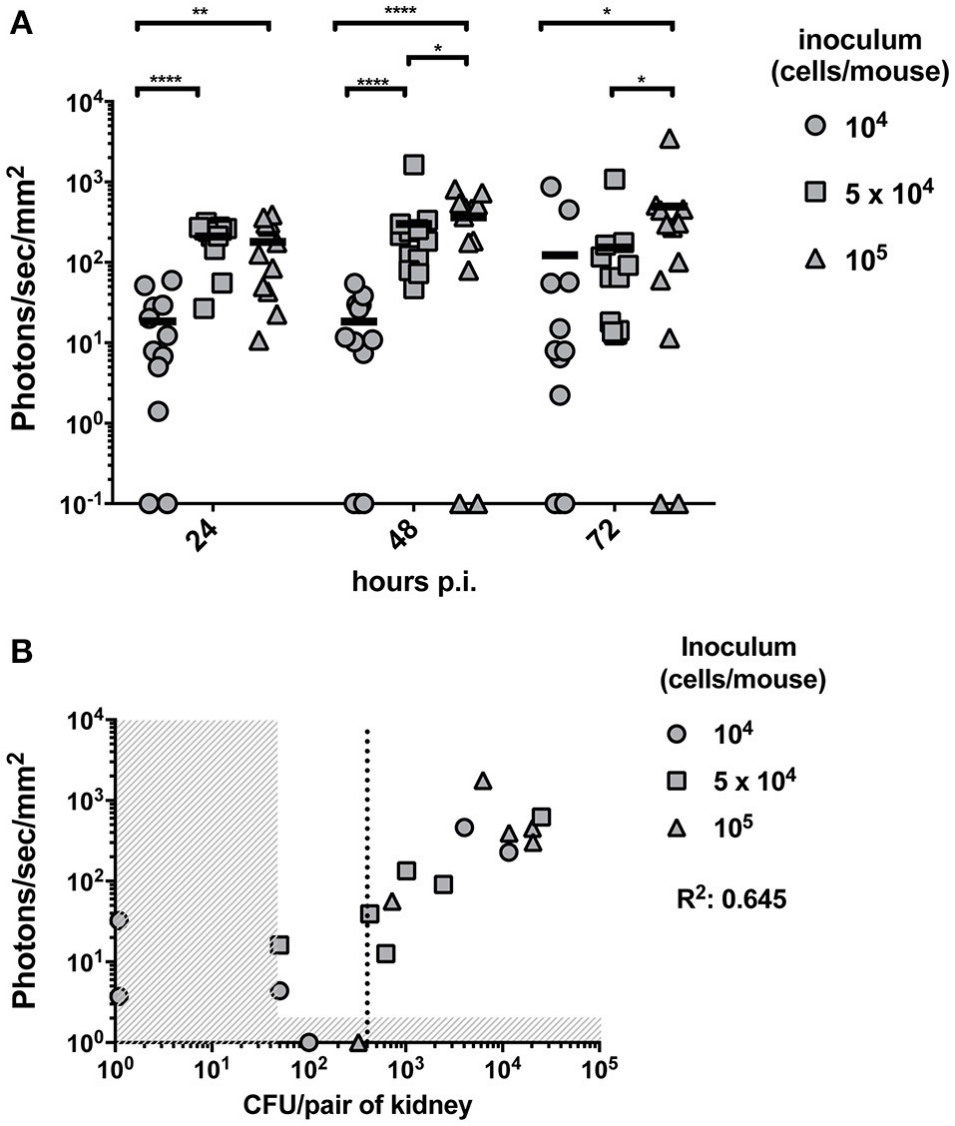
*In vivo* dose-response experiment and determination of the limit of detection. **(A)** Dose response experiment was performed by injecting 10^4^, 5 × 10^4^ or 10^5^ and the luminescent signal was followed at 24-, 48-, and 72 h p.i. (*n* = 6 mice /group). Background signal was subtracted from the individual kidney signals. Corrected signals are represented in photon/s/mm^2^. Kidney signals below background are plotted on the X-axis and were excluded for statistical analysis (Mann-Whitney unpaired test, *p*-value: ^*^ < 0.05, ^**^ < 0.01, ^****^ < 0.0001). **(B)** For each individual animal, the CFU/g measured from both kidneys at 72 h p.i. is plotted against the average kidney luminescent signal. The detection thresholds (50 CFU/pair of kidneys and 10 photons/s/mm^2^) are indicated by the vertical and horizontal dashed regions, respectively. A linear regression calculated on the detectable measures was performed and the *R*^2^ value is indicated. A dotted line indicates the detection limit of CFU based on the detection threshold of luminescence.

In this part of the study we aimed to establish the limit of detection of Mut2 *in vivo*. For this purpose, mice were infected with three different inoculums (10^4^, 5 × 10^4^, and 10^5^ cells/mice) of *C. albicans* strain expressing Mut2. Infection was monitored daily for three consecutive days recording kidney luminescence emission (Figure 3A). Globally, kidney signals were stable between 24- and 72 h p.i. A dose response was visible with a statistical significant difference between mice infected with 10^4^ cells as compared to mice infected with 10^5^ cells at the three time-points. The infection dose of 10^4^ cells gave more variable outcomes of infection (Figure 3A).

To establish the limit of detection, mice were sacrificed at 72 h p.i.. Fungal burdens were measured and correlated with the corresponding previously recorded luminescent signals. Due to technical limitations, it is not possible to detect <50 CFU per pair of kidney. Luminescence signals above the background detection levels were considered as bioluminescent signals produced by *C. albicans*. Both thresholds are indicated in Figure 3B by dashed regions. The detection limit should be set above the two thresholds. Such limit was estimated at about 400 CFU/pair of kidney (dotted line of Figure 3B), which corresponds to about 200 CFU/kidney.

### Kinetics of mice systemic infection

**Figure 4:**
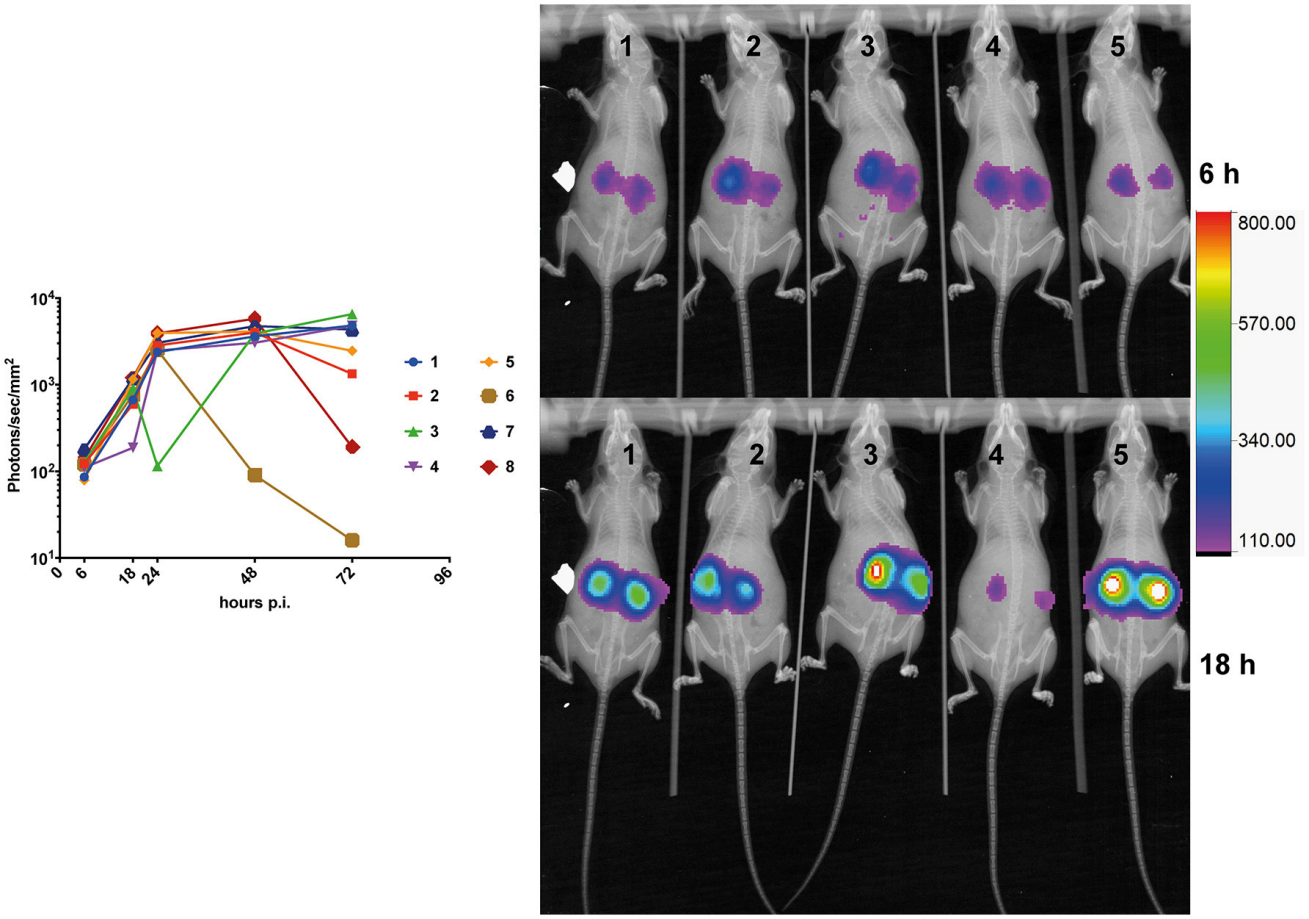
Kinetics of mouse kidney infection. The average kidney luminescent signal, recorded at 6-, 18-, 24-, and 48 h p.i. (5 × 10^5^ cells/mouse) is represented and each mouse signal is connected overtime.

Kinetics of bioluminescence emission was addressed in infected mice (*n* = 8) at early (6-, 18-, and 24 h) and later time points (48- and 72 h). Light emission was originating mainly from the region of the kidneys. While a constant progression of emitted light was observed up to 24 h p.i. in almost all animals (Figure 4), signals tended to stabilize at the later time points as observed previously in Figure 3A, and dropped in one individual (Figure 4, animal No 6). This observation suggests that fungal loads increase dramatically in infected kidneys in the first 24 h p.i., and stabilized thereafter. This is consistent with observations made by Jacobsen et al. (2014), who suggested that fungal loads were principally increasing over 24 h p.i.

### *In vivo* monitoring of treatment efficacy on *C. albicans* systematic infection in mice

BLI was next used to evaluate the efficacy of antifungal treatments on *C. albicans* (Mut2) infections initiated by intravenous challenge. We used fluconazole (FLC) and caspofungin (CAS) as therapeutic agents in these experiments. Infected animal treatments were started intraperitoneally 24 h after inoculation, and then administrated daily. Animals were individually followed by BLI for a period of 72–96 h p.i..

**Figure 5:**
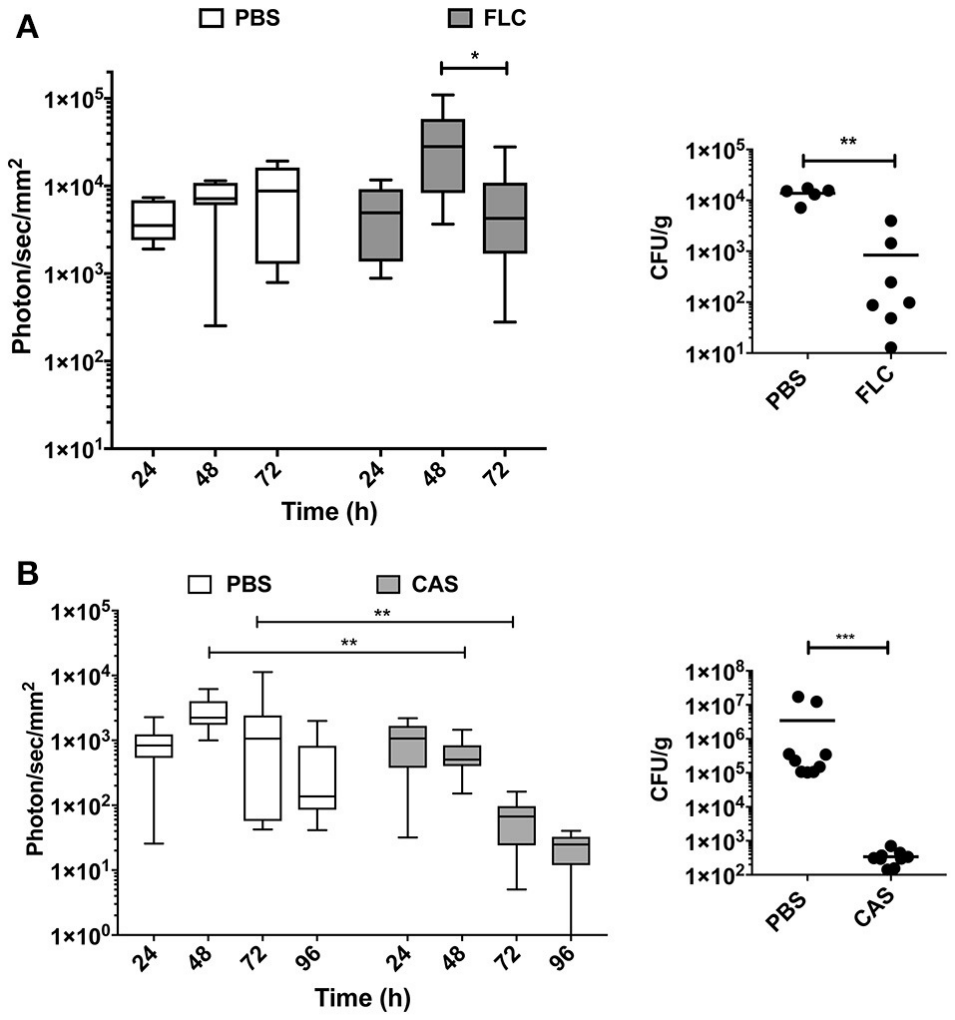
*In vivo* dynamics of infection during drug treatment. Drug treatment was initiated 24 h p.i. [intraperitoneal injection of fluconazole (FLC): 10 mg/kg **(A)**, caspofungin (CAS): 1 mg/kg **(B)**] and repeated at 48 h p.i. for FLC and 48 h p.i., and 96 h p.i. for CAS. (Mann-Whitney unpaired test, *p*-value: ^*^ < 0.05, ^**^ < 0.01, ^****^ < 0.0001).

In the fluconazole experiment, the mean luminescence of untreated control animals increased ~2-fold over a 72 h period of treatment (Figure 5A). In fluconazole-treated animals, bioluminescence increased up to 48 h and then declined. These data suggest a delay in fluconazole efficacy. Since fluconazole was injected 24 h p.i., this delay may reflect limitations in the pharmacokinetics of this antifungal agent. The fluconazole effect observed with luminescent signals could be confirmed measuring CFU at 72 h p.i. toward end of the experiment. While untreated animals had a fungal burden around 10^4^ CFU/g of kidney, treated animals displayed 10- to 100 times lower kidney fungal burden (Figure 5A, right panel).

The caspofungin treatment experiment was performed separately and included an independent untreated control group. Figure 5B indicates that bioluminescence signals of the untreated group were first increasing over 72 h and then declined at 96 h p.i. In contrast, caspofungin-treated mice showed a decreasing bioluminescence all along the entire treatment time course, particularly from 48 to 96 h p.i. Efficacy of caspofungin treatment could be confirmed with fungal burden of kidneys in infected animals at 96 h: while CFUs were slightly above the limit of detection in treated animals (10^2^/g kidneys), fungal burdens were ranging between 10^5^ and 10^7^ CFUs/g kidney in untreated animals. These experiments clearly highlight differences in the pharmacodynamics of fluconazole and caspofungin treatments.

### *In vivo* monitoring of *C. albicans* OPC infection in mice

**Figure 6:**
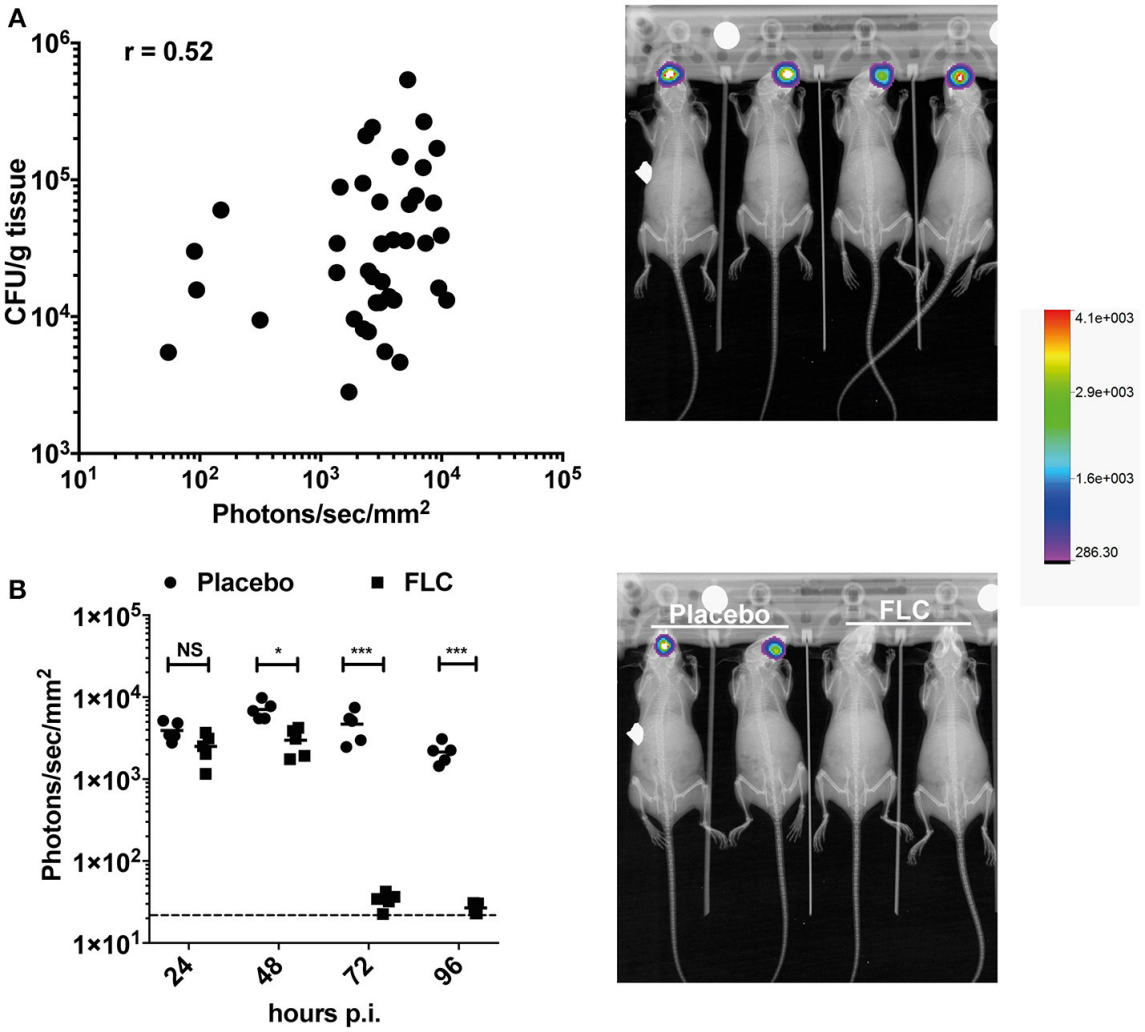
BLI in the *C. albicans* OPC model. **(A)** Correlation of BLI with CFUs of infected tongues. Data are originating from several independent infection experiments with the *C. albicans* persistent isolate 101 expressing Mut2 (DSY4976). CFUs from homogenized tongues were taken at 96 h p.i.. In the right panel, BLI from in-vivo Xtreme II (overlay of X-ray and bioluminescence capture) is shown to highlight the region of light emission. **(B)** FLC treatment of mice in the OPC model. Data show bioluminescence signals obtained from untreated (Placebo, *n* = 6) and FLC-treated animals (*n* = 6). Mice were infected as described in Section Material and Methods. FLC treatment was initiated at 24 p.i. with oral application of FLC (10 mg/kg) and repeated daily until sacrifice. In the right panel, BLI from in-vivo Xtreme II (overlay of BLI on X-ray) is shown to highlight the location of light emission in untreated and treated animals. (Mann-Whitney unpaired test, *p*-value: ^*^ < 0.05, ^****^ < 0.0001, NS, not significant).

The OPC model was also tested with bioluminescent *C. albicans* cells expressing Mut2. To assess this model of infection with a bioluminescence read-out, we used a persistent *C. albicans* clinical isolate (strain 101). This strain is reported as persistent in the OPC model of infection as opposed to the strain SC5314, which is known to be cleared rapidly in this infection model (Schönherr et al., 2017). In our hands, strain 101 can persist for at least 96 h in the OPC model (Figure 6B), while SC5314 is cleared within 3 days p.i. (data not shown). Infecting mice with the strain 101 expressing Mut2 in the oropharyngeal area resulted as expected in luminescence signals located in the head region (Figures 6A,B). We used several experiments in which CFUs were obtained from animal's tongues at the end of experimentation (96 h) to correlate oral cavity luminescent signal with tongue fungal burden. Figure 6A shows that bioluminescence signals were correlated to recovered CFUs. The correlation was significant, even though the correlation coefficient (*R* = 0.52) was not excellent. We next addressed the efficacy of FLC in this model by applying the substance sublingual at three different time points post infection (24-, 48-, and 72 h). While a significant decrease in light emission was detected starting at 48 h post-treatment as compared to placebo, no light was detected in FLC-treated at later time points (up to 96 h; Figure 6B). In agreement, no *C. albicans* cells were recovered from tongues of treated animals at 96 h post-treatment (data not shown). These data clearly demonstrate the utility of BLI in this type of infection model.

## Discussion

In this work, we designed a codon optimized luciferase with red-shifted properties. Earlier work performed with firefly luciferase adapted to mammalian expression identified a mutation (S284T) causing a red-shift (Branchini et al., 2005). The introduction of this mutation in the *C. albicans* codon-optimized luciferase was accompanied by addition of other mutations described as conferring thermal stability and enhanced light emitting activity (L295F, T214A, and A215L). The resulting protein (Mut2) expectedly produced efficient red-shifted light *in vitro* and *in vivo*. We next attempted to modify the firefly luciferase Mut2 by other thermal stability mutations (I232A, I351V) and further red-shifting (F465R) as previously described (Branchini et al., 2010; see Figure 1A for details). However, the resulting proteins did not confer better properties as compared to Mut2 at least *in vitro* (data not shown). The introduction of these last mutations in the codon-optimized firefly luciferase, and particularly in Mut2, may result in improper folding and destabilization. As published by Branchini et al. (2010), these effects need to be compensated by the introduction of additional mutations. Besides these protein modifications, it may be valuable in the future to introduce into *C. albicans* the genes responsible for the production of luciferin, which is the substrate of firefly luciferase. This will avoid the need to inject luciferin repeatedly, and will also overcome any concerns regarding luciferin diffusion into infected tissues. This has been achieved in mammalian cells which thus became autonomous for light emission (Close et al., 2010).

BLI has been used already in *C. albicans* in different studies. A codon-optimized firefly luciferase was first used in *C. albicans* and analyzed *in vivo* with systemic and vaginal infection model (Doyle et al., 2006a,b). While the relationship between signal intensity and CFU was satisfactory in the vaginal infection model, the authors concluded that emitted signals were too low in the invasive infection model and that there was a lack of correlation between emitted light and fungal loads in this model (Doyle et al., 2006a). Some of the signal detection issues of this study could have been caused by first generation BLI equipments with poor detection limits.

Later, firefly luciferase was also used by Jacobsen et al. (2014) in *C. albicans*, however, light detection was performed with a more recent instrumentation. In this study, Jacobsen et al. (2014) successfully detected *C. albicans* bioluminescent signals and established that this signal highly correlated with the fungal loads. Authors estimated that the sensitivity of the codon optimized firefly luciferase was adequate for visualization of the infection (Jacobsen et al., 2014).

We thus attempted to compare the performance of BLI in the present study with that published using the systemic infection model and light detection in the region of the kidneys. We estimated that a lower limit of detection for BLI (50 photon/s/mm^2^) corresponded to of about 200 CFU/kidney with the instrumentation used here (in-vivo Xtreme II). Given numbers published by Jacobsen et al. (2014) (100 photon/s/mm^2^ for 3,600 CFU/kidney), this suggests that the method used here increases at least by 4- to 8-fold the sensitivity of *C. albicans* detection. This performance can be attributed to several factors including (i) the type of luciferase used (Mut2 has better tissue penetration capacities than the wild type luciferase) and (ii) technical differences provided by the luminescence acquisition of instruments. This performance is even more remarkable when taking in account that BLI data was collected here from animals contained in Sealed OI Trays, which may create another obstacle of emitted light to the high sensitivity camera.

We used BLI to establish two animal models of *C. albicans* infection (systemic and OPC model). The systemic model has been used by others, as mentioned above, with different outcomes. The OPC model in immunocompromised mice was used recently but with a *Gaussia* luciferase expressed at the surface of *C. albicans* cells (Gabrielli et al., 2015). The use of this luciferase system is possible for surface mucosal *C. albicans* infection but is confronted by limitations in deeper tissue infections due to poor penetration of blue emitted light from the *Gaussia* luciferase. It is difficult to compare the performance of the two systems since different equipments and image acquisitions parameters were used. The *Gaussia* luciferase uses coelenterazine as a substrate for light emission and this substance is known to produce background luminescence in animals, thus causing problems in data quantification especially at low fungal burden (Vande Velde et al., 2013; Gabrielli et al., 2015). The use here of the modified firefly luciferase (Mut2) offers an attractive alternative to this model due to a low signal/noise ratio.

In conclusion, we presented here an efficient and non-invasive method to assess fungal burden in *C. albicans* infected animals. This system takes advantage of a modified firefly luciferase and of an efficient and sensitive image capture equipment, the in-vivo Xtreme II. This system may be implemented in the future for other fungal pathogens and animal models to study fungal-host interactions.

## Author contributions

SD and AC performed experiments and participated to manuscript writing. DS designed experiments and participated to manuscript writing.

### Conflict of interest statement

The authors declare that the research was conducted in the absence of any commercial or financial relationships that could be construed as a potential conflict of interest.

## Supplementary material

Figure S1 Immunodetection of firefly luciferase in *C. albicans*.

Movie S1 Workflow of bioluminescence imaging with in-vivo Xtreme II. Mice used in this experiment were first infected by *C. albicans* by tail intravenous injection. Before imaging, shaved mice were placed in an induction chamber with constant isofluorane flow.

## References

[B1] Amorim-Vaz S. Delarze E. Ischer F. Sanglard D. Coste A. T. (2015). Examining the virulence of *Candida albicans* transcription factor mutants using *Galleria mellonella* and mouse infection models. Front. Microbiol. 6:367. 10.3389/fmicb.2015.00367 25999923 PMC4419840

[B2] Barelle C. J. Manson C. L. MacCallum D. M. Odds F. C. Gow N. A. R. Brown A. J. P. (2004). GFP as a quantitative reporter of gene regulation in *Candida albicans*. Yeast 21, 333–340. 10.1002/yea.1099 15042593

[B3] Branchini B. R. Ablamsky D. M. Davis A. L. Southworth T. L. Butler B. Fan F.. (2010). Red-emitting luciferases for bioluminescence reporter and imaging applications. Anal. Biochem. 396, 290–297. 10.1016/j.ab.2009.09.009 19748472

[B4] Branchini B. R. Ablamsky D. M. Murtiashaw M. H. Uzasci L. Fraga H. Southworth T. L. (2007). Thermostable red and green light-producing firefly luciferase mutants for bioluminescent reporter applications. Anal. Biochem. 361, 253–262. 10.1016/j.ab.2006.10.043 17181991

[B5] Branchini B. R. Southworth T. L. Khattak N. F. Michelini E. Roda A. (2005). Red- and green-emitting firefly luciferase mutants for bioluminescent reporter applications. Anal. Biochem. 345, 140–148. 10.1016/j.ab.2005.07.015 16125663

[B6] Brock M. (2012). Application of bioluminescence imaging for *in vivo* monitoring of fungal infections. Int. J. Microbiol. 2012:956794 10.1155/2012/956794 PMC3205719 22121368

[B7] Brown G. D. Denning D. W. Gow N. A. R. Levitz S. M. Netea M. G. White T. C. (2012). Hidden killers: human fungal infections. Sci. Transl. Med. 4:165rv13. 10.1126/scitranslmed.3004404 23253612

[B8] Close D. M. Patterson S. S. Ripp S. Baek S. J. Sanseverino J. Sayler G. S. (2010). Autonomous bioluminescent expression of the bacterial luciferase gene cassette (lux) in a mammalian cell line. PLoS ONE 5:e12441. 10.1371/journal.pone.0012441 20805991 PMC2929204

[B9] Coste A. T. Amorim-Vaz S. (2015). Animal models to study fungal virulence and antifungal drugs, in Antifungals: From Genomics to Resistance and the Development of Novel Agents, eds Coste A. T. Vandeputte P. (Norfolk, UK: Caister Academic Press), 289–316.

[B10] Doyle T. C. Nawotka K. A. Kawahara C. B. Francis K. P. Contag P. R. (2006a). Visualizing fungal infections in living mice using bioluminescent pathogenic *Candida albicans* strains transformed with the firefly luciferase gene. Microb. Pathog. 40, 82–90. 10.1016/j.micpath.2005.11.003 16426810

[B11] Doyle T. C. Nawotka K. A. Purchio A. F. Akin A. R. Francis K. P. Contag P. R. (2006b). Expression of firefly luciferase in *Candida albicans* and its use in the selection of stable transformants. Microb. Pathog. 40, 69–81. 10.1016/j.micpath.2005.11.002 16427765

[B12] Enjalbert B. Rachini A. Vediyappan G. Pietrella D. Spaccapelo R. Vecchiarelli A.. (2009). A multifunctional, synthetic *Gaussia princeps* luciferase reporter for live imaging of *Candida albicans* infections. Infect. Immun. 77, 4847–4858. 10.1128/IAI.00223-09 19687206 PMC2772526

[B13] Fonzi W. Irwin M. (1993). Isogenic strain construction and gene mapping in *Candida albicans*. Genetics 134, 717–728. 8349105 10.1093/genetics/134.3.717 PMC1205510

[B14] Gabrielli E. Roselletti E. Luciano E. Sabbatini S. Mosci P. Pericolini E. (2015). Comparison between bioluminescence imaging technique and CFU count for the study of oropharyngeal candidiasis in mice. Cytometry A 87, 428–436. 10.1002/cyto.a.22666 25820122

[B15] Jacobsen I. D. Lüttich A. Kurzai O. Hube B. Brock M. (2014). *In vivo* imaging of disseminated murine *Candida albicans* infection reveals unexpected host sites of fungal persistence during antifungal therapy. J. Antimicrob. Chemother. 69, 2785–2796. 10.1093/jac/dku198 24951534

[B16] Kapitan M. Eichhof I. Lagadec Q. Ernst J. F. (2016). Click beetle luciferases as dual reporters of gene expression in *Candida albicans*. Microbiology 162, 1310–1320. 10.1099/mic.0.000329 27339610

[B17] MacCallum D. M. Odds F. C. (2005). Temporal events in the intravenous challenge model for experimental *Candida albicans* infections in female mice. Mycoses 48, 151–161. 10.1111/j.1439-0507.2005.01121.x 15842329

[B18] Mosci P. Pericolini E. Gabrielli E. Kenno S. Perito S. Bistoni F.. (2013). A novel bioluminescence mouse model for monitoring oropharyngeal candidiasis in mice. Virulence 4, 250–254. 10.4161/viru.23529 23334179 PMC3711983

[B19] Murad A. M. Lee P. R. Broadbent I. D. Barelle C. J. Brown A. J. (2000). CIp10, an efficient and convenient integrating vector for *Candida albicans*. Yeast 16, 325–327. 10.1002/1097-0061(20000315)16:4<325::AID-YEA538>3.0.CO;2-# 10669870

[B20] Papon N. Courdavault V. Lanoue A. Clastre M. Brock M. (2014). Illuminating fungal infections with Bioluminescence. PLoS Pathog. 10:e1004179. 10.1371/journal.ppat.1004179 25010008 PMC4092138

[B21] Reuss O. Vik Å. Kolter R. Morschhäuser J. (2004). The SAT1 flipper, an optimized tool for gene disruption in *Candida albicans*. Gene 341, 119–127. 10.1016/j.gene.2004.06.021 15474295

[B22] Sanglard D. Ischer F. Monod M. Bille J. (1996). Susceptibilities of *Candida albicans* multidrug transporter mutants to various antifungal agents and other metabolic inhibitors. Antimicrob. Agents Chemother. 40, 2300–2305. 8891134 10.1128/aac.40.10.2300 PMC163524

[B23] Schönherr F. A. Sparber F. Kirchner F. R. Guiducci E. Trautwein-Weidner K. Gladiator A. (2017). The intraspecies diversity of *C. albicans* triggers qualitatively and temporally distinct host responses that determine the balance between commensalism and pathogenicity. Mucosal Immunol. 85:e0080 10.1038/mi.2017.2 28176789

[B24] Sturtevant J. (2009). Reporter gene assays in *Candida albicans*. Methods Mol. Biol. 499, 157–167. 10.1007/978-1-60327-151-6_15 19152047

[B25] Trautwein-Weidner K. Gladiator A. Kirchner F. R. Becattini S. Rülicke T. Sallusto F.. (2015). Antigen-Specific Th17 Cells Are Primed by distinct and complementary Dendritic Cell Subsets in Oropharyngeal Candidiasis. PLoS Pathog. 11:e1005164. 10.1371/journal.ppat.1005164 26431538 PMC4591991

[B26] Vande Velde G. Kucharíková S. Schrevens S. Himmelreich U. Van Dijck P. (2013). Towards non-invasive monitoring of pathogen-host interactions during *Candida albicans* biofilm formation using *in vivo* bioluminescence. Cell. Microbiol. 16, 115–130. 10.1111/cmi.12184 23962311 PMC4204156

[B27] Zhang J.-E. Luo D. Chen R.-Y. Yang Y.-P. Zhou Y. Fan Y.-M. (2013). Feasibility of histological scoring and colony count for evaluating infective severity in mouse vaginal candidiasis. Exp. Anim. 62, 205–210. 10.1538/expanim.62.205 23903055 PMC4160942

