# Supplementary material for: Red-Shifted Firefly Luciferase Optimized for Candida albicans In vivo Bioluminescence Imaging
Source: Front Microbiol. 2017 Aug 3;8:1478. doi: 10.3389/fmicb.2017.01478 (PMC5541039; doi:10.3389/fmicb.2017.01478)
Supplement: Figure S1 — Immunodetection of firefly luciferase in C. albicans. [file Image1.PDF]

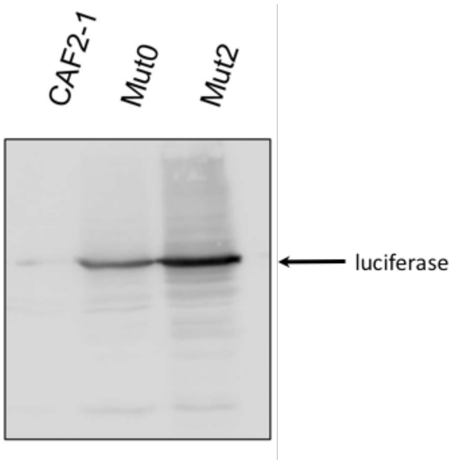

Figure S1: Immunodetection of firefly luciferase in *C. albicans*. For protein extractions, an alkaline extraction procedure was used as described (Sanglard et al., 1998). Cells were grown in YEPD medium overnight. Five milliliters of YEPD medium was inoculated with 100 µl of 10^8 cells/ml, and cells were grown to a concentration of 1.5×10^7 cells/ml. The samples were separated in a 10% (wt/vol) polyacrylamide gel. The gel was transferred onto a nitrocellulose membrane by Western blotting. Firefly luciferase was then detected by chemoluminescence (ECL kit; Amersham, Bioscience) using polyclonal rabbit anti-luciferase antibodies and an anti-rabbit horseradish peroxidase-labeled secondary antibody. Origins of extract are shown as indicated. Protein extract of strain CAF2-1 served as control (Fonzi and Irwin, 1993).

Reference

Fonzi, W. A., and Irwin, M. Y. (1993). Isogenic strain construction and gene mapping in *Candida albicans*. *Genetics* 134, 717–728.

Sanglard, D., Ischer, F., Koymans, L., and Bille, J. (1998). Amino acid substitutions in the cytochrome P-450 lanosterol 14alpha-demethylase (CYP51A1) from azole-resistant *Candida albicans* clinical isolates contribute to resistance to azole antifungal agents. *Antimicrob Agents Chemother* 42, 241–253.
